# Antimicrobial Resistance Genes and Clonal Relationships of Duck-Derived *Salmonella* in Shandong Province, China in 2023

**DOI:** 10.3390/microorganisms12122619

**Published:** 2024-12-18

**Authors:** Zhiyuan Lu, Yue Zheng, Shaopeng Wu, Xiaoyue Lin, Huiling Ma, Xiaofei Xu, Shumin Chen, Jiaqi Huang, Zheng Gao, Guisheng Wang, Shuhong Sun

**Affiliations:** 1 Shandong Provincial Key Laboratory of Zoonoses, College of Animal Medicine, Shandong Agricultural University, Tai’an 271002, China; 2022110423@sdau.edu.cn (Z.L.); wsp@sdau.edu.cn (S.W.); 2020110516@sdau.edu.cn (J.H.); 2 State Key Laboratory of Crop Biology, College of Life Sciences, Shandong Agricultural University, Tai’an 271002, China; 2022010158@sdau.edu.cn (Y.Z.); gaozheng@sdau.edu.cn (Z.G.); 3 Shandong Provincial Center for Animal Disease Control, Jinan 250100, China; linxiaoyue@shandong.cn (X.L.); 18553159555@163.com (H.M.); 18853195892@163.com (X.X.); csm7034@sina.com (S.C.)

**Keywords:** *Salmonella*, whole genome sequencing, sequence types, duck, antibiotic resistance genes, mutation

## Abstract

*Salmonella* is a major threat to both human and animal health. However, the diversity and antibiotic resistance of animal-derived *Salmonella* and their association with human infections remain largely unexplored. In this study, *Salmonella* strains were isolated, identified, and sequenced from dead embryos and cloacal swab samples obtained from 278 large-scale duck farms in 11 cities in Shandong Province. The results show that a total of 57 *Salmonella* strains were isolated, with the dominant sequence types (ST) being ST17 (15/57) and ST19 (9/57), while the dominant serotypes were *S. Indiana* (15/57) and *S. Typhimurium* (11/57). Furthermore, genomic analysis has revealed the presence of prevalent antibiotic resistance genes (ARGs), which are often associated with co-transfer mechanisms. Over 52.63% of the strains were observed to carry two or more ARGs, especially one *Salmonella* strain that carried twenty-eight distinct ARGs. Furthermore, core genome multilocus sequence typing analysis (cgMLST) indicated that the 57 *Salmonella* strains may have a close relationship, which could be clonally transmitted among different cities. The results demonstrated a close relationship between the *Salmonella* strains identified in diverse geographical regions, suggesting that these strains may have been widely disseminated through clonal transmission. The mutation analysis reveals significant mutations at *parC* (T57S), *gyrA* (S83F), *parC* (S80R), *gyrA* (D87N), and *gyrA* (S83Y). These findings emphasize the necessity for monitoring and controlling *Salmonella* infections in animals, as they may serve as a reservoir for ARGs with the potential to affect human health or even be the source of pathogens that infect humans.

## 1. Introduction

*Salmonella* is a zoonotic bacterial threat to global public health and one of the most prevalent foodborne pathogens [1]. In addition to the economic losses incurred on the farm, concerns about the zoonotic potential of *Salmonella* may also give rise to economic effects. The primary means of transmission of *Salmonella* is via the fecal-oral route [2]. Infected animals may shed bacteria, either asymptomatically or during and after clinical illness, thereby contaminating the environment or infecting others directly. The spread of *Salmonella* between flocks can be influenced by several factors, including trade, contact with other flocks, use of infected water, lack of adequate biosecurity measures for visitors, and sharing of equipment. It is important to note that animals, including chickens, ducks, swine, cattle, and some domestic companion animals, have not escaped the threat of *Salmonella* [3,4]. For example, *Salmonella* enterica serovar Pullorum has the potential to cause salmonellosis in poultry, resulting in huge losses in poultry production. The contamination of poultry products with *Salmonella* represents a significant concern due to its potential to cause severe economic losses and public health problems [5]. The objective of the “one health” approach is to achieve a state of equilibrium and optimality in the health of human beings, animals, and ecosystems [6,7].

Bacterial antimicrobial resistance (AMR) is considered to be an important factor in the increase of human mortality worldwide [8], and the widespread use of antibiotics in the poultry industry can easily lead to the emergence of multidrug-resistant (MDR) *Salmonella* [9]. The pervasive use of antibiotics in poultry and animal husbandry is a matter of particular concern because antibiotic resistance is widespread in *Salmonella* strains from poultry [3,10]. The indiscriminate and unregulated use of antibiotics has led to an increase in the prevalence of MDR *Salmonella* in animals and humans, and MDR *Salmonella* can be transmitted to humans through animal products or the environment, posing significant public health and food safety concerns [4,11]. With increasing awareness of the evolution of AMR in *Salmonella*, governments and researchers have initiated longitudinal surveillance programs, which have yielded valuable epidemiological data for risk assessment and medication guidelines [12].

Bacterial typing is an essential method for bacterial identification, epidemiological investigation, traceability, and food safety monitoring, which is of great importance to food safety and public health [13]. However, whole genome sequencing (WGS) may prove to be more beneficial as it allows the core genome to be assessed by SNP-based or MLST methods, which can determine evolutionary relationships. Furthermore, WGS can facilitate the identification of AMR determinants and the prediction of phenotypic antibiotic resistance spectra [14]. As a result, affordable WGS has become a widely used molecular typing technology for tracking *Salmonella* transmission [15,16].

Ducks are important waterfowl that are widely reared in China, and they can spread *Salmonella* through the food supply chain [17]. Shandong Province has the highest production of poultry products (including ducks) in China [18]. Duck is an important host for *Salmonella*. As with other types of meat products, consumption of contaminated duck meat will lead to *Salmonella* infection. In recent years, duck meat has been widely accepted by consumers due to its sensory characteristics, high concentration of unsaturated fatty acids, and other nutritional factors. However, the existing research still lacks a comprehensive understanding of the diversity and drug resistance determinants of Salmonella from ducks. This study is primarily concerned with duck-derived *Salmonella* for two main objectives. The first is to elucidate the diversity and dynamics of duck-derived *Salmonella* in order to reduce the economic losses caused by these organisms. The second is to determine the distribution of ARGs in duck-derived *Salmonella*. In this study, we sequenced the large-scale genome of *Salmonella* isolated from duck embryo samples and cloacal swabs from 278 duck farms in 11 cities of Shandong Province, China, in 2023. The relationships between the population structure, ARGs, and genotypes of duck-derived *Salmonella*, and the potential impact of these *Salmonella* on human infection were also considered.

## 2. Materials and Methods

### 2.1. Main Reagents

Buffered peptone water (BPW), tetrathionate broth (TTB)-enriched broth, selenocysteine (SC)-enriched broth, Rappaport Vassiliadis *Salmonella* (RVS)-enriched broth, and xylose-lysine deoxycholate (XLD) were purchased from HaiBo BioTech Ltd. (Qingdao, China).

### 2.2. Salmonella Isolates Collection

The *Salmonella* isolates were collected from 325 dead embryos and 13,260 cloacal swab samples of ducks from 278 duck farms in 11 cities in Shandong Province by the Shandong Provincial Center for Animal Disease Control in 2023. The dead embryo in the eggshell was then removed, and the liver, spleen, intestinal segment, and yolk samples were aseptically transferred to the separately packaged BPW enrichment solution and sample retention centrifuge tube. Each dead embryo and cloacal swab sample was added to 4.5 mL of BPW and then incubated at 37 °C for 12 h for pre-enrichment. Approximately 0.5 mL of pre-enriched culture was inoculated into 4.5 mL of TTB, SC, and RVS, respectively. Cultures of each TTB, SC, and RVS broth were inoculated on an XLD Agar base and incubated at 37 °C for 48 h.

### 2.3. DNA Extraction and PCR

Genomic DNA was extracted from all *Salmonella* isolates using a commercial DNA kit (TIANGEN, Beijing, China). The quality and concentration of the bacterial genomic DNA were assessed by electrophoresis on a 1% agarose gel, and analysis was conducted using a NanoDrop2000 system (Thermo Scientific, Waltham, MA, USA) and a Qubit 4 fluorometer (Thermo Scientific, Waltham, MA, USA). The confirmation of smooth and round colonies without a black center or large colonies with a black center was achieved by polymerase chain reaction (PCR) assays with primers designed for the *FimW* gene [3].

### 2.4. WGS

The bacteria were sent to Novogene Biology Science and Technology Co., LTD (Tianjin, China) for genome sequencing. The original data of the WGS were spliced by quality control (https://enterobase.warwick.ac.uk/, accessed on 15 January 2024) and then uploaded to the website of Center for Genomic Epidemiology (CGE) for partial bioinformatics analysis [19]. Among them, the tool (https://cge.food.dtu.dk/services/SerotypeFinder/, accessed on 9 April 2024) was used to analyze the serotype of *Salmonella*; Using ResFinder 4.1 tool (https://cge.food.dtu.dk/services/ResFinder/, accessed on 12 April 2024) to select parameters of 60% minimum length and 90% threshold to match the strain gene with the database resistance gene for acquired drug resistance gene analysis [20].

### 2.5. Genome Annotation and Analysis

The raw reads were uploaded to the EnteroBase *Salmonella* database (https://enterobase.warwick.ac.uk/, accessed on 25 April 2024), which contains the results of legacy multilocus sequence typing and core genome MLST (cgMLST). Meanwhile, the software SPAdes_3.12.0 was used to assemble draft genomes [21]. The prokaryotic genome annotation website RAST (http://rast.nmpdr.org/, accessed on 5 July 2024) was used to obtain the basic information on the draft genomes. The genome sequences were analyzed for plasmid replicons, ARGs, and mutation sites using the CGE PlasmidFinder database 2.0.1, ResFinder database v2.0, ABRicate vs. 0.8, and RGI-CARD vs. 3.0.

## 3. Results

### 3.1. Isolation of Salmonella Strains

**Figure 1:**
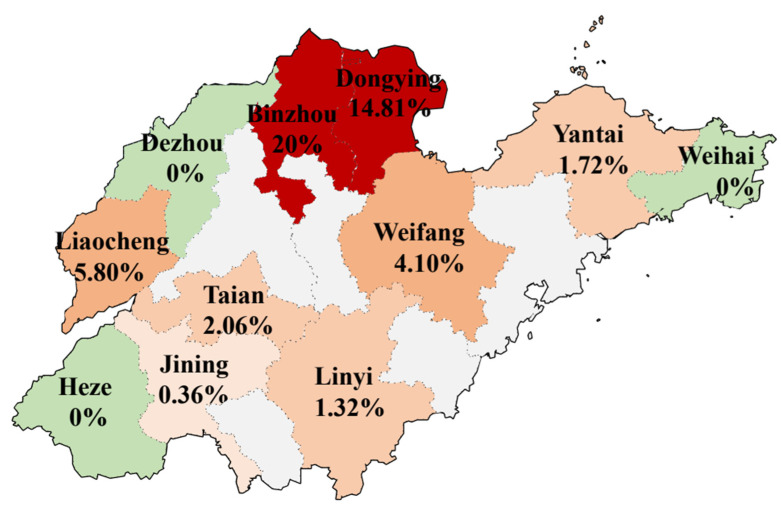
The geographical distribution of 11 cities sampled in Shandong Province and the positive rate of *Salmonella* from ducks. The scale is 1:400,000.

A total of 57 *Salmonella* strains were isolated from 13585 samples collected from 278 duck farms in Shandong Province in 2023. As shown in Figure 1, among the 11 cities in Shandong Province, the positive rates of *Salmonella* were highest in Binzhou and Dongying, reaching 20% and 14.81%, respectively. In contrast, the positive rates of *Salmonella* were less than 6% in Liaocheng, Weifang, Tai’an, Yantai, Linyi, and Jining, and no *Salmonella* was detected in Dezhou, Heze, and Weihai. The above results show that the isolation rate of *Salmonella* in Shandong Province in 2023 is different in different cities.

### 3.2. STs Detection

**Figure 2:**
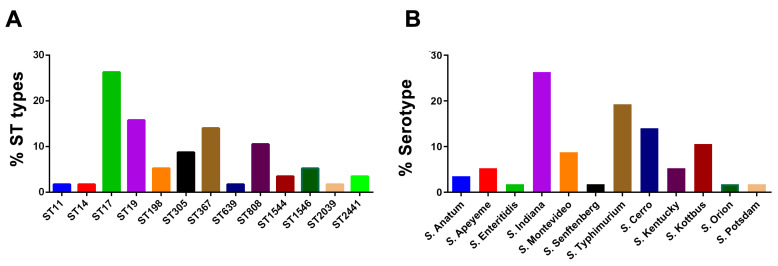
Prevalence of STs and serotypes in *Salmonella* strains. (**A**) STs. (**B**) serotypes.

As shown in Figure 2A, the STs classification showed that the 57 *Salmonella* strains fell into 13 STs: ST11 (1/57, 1.75%), ST14 (1/57, 1.75%), ST17 (15/57, 26.32%), ST19 (9/57, 15.79%), ST198 (3/57, 5.26%), ST305 (5/57, 8.77%), ST367 (8/57, 14.04%), ST639 (1/57, 1.75%), ST808 (6/57, 10.53%), ST1544 (2/57, 3.51%), ST1546 (3/57, 5.26%), ST2039 (1/57, 1.75%), and ST2441 (2/57, 3.51%) (Figure 2A). Importantly, the dominant ST was ST17.

### 3.3. Serotypes Detection

As shown in Figure 2B, serotype detection showed that the 57 *Salmonella* strains fell into 12 different serotype types: *S. Anatum* (2/57, 3.51%), *S. Apeyeme* (3/57, 5.26%), *S. Enteritidis* (1/57, 1.75%), *S. Indiana* (15/57, 26.32%), *S. Montevideo* (5/57, 8.77%), *S. Senftenberg* (1/57, 1.75%), *S. Typhimurium* (11/57, 19.30%), *S. Cerro* (8/57, 14.04%), *S. Kentucky* (3/57, 5.26%), *S. Kottbus* (6/57, 10.53%), *S. Orion* (1/57, 1.75%) and *S. Potsdam* (1/57, 1.75%). Notably, the dominant serotype is *S. Indiana*. The results showed that except for ST19 and ST1544, which belonged to *S. Typhimurium*, there was a one-to-one relationship between the other *Salmonella* serotypes and STs.

### 3.4. Detection of ARGs

**Figure 3:**
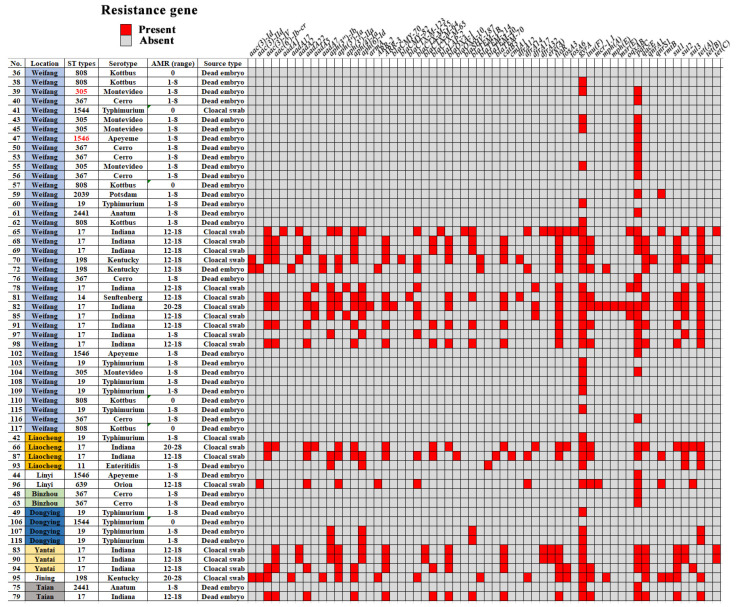
Specific information of *Salmonella* strains, including serial number, location, STs, serotype, ARG range, source type, and drug resistance gene.

**Figure 4:**
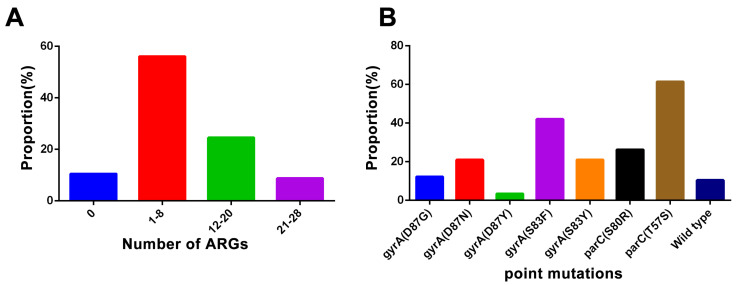
Prevalence of ARGs and frequency of mutations of ARGs (*gyrA* and *parC*) in *Salmonella* strains. (**A**) The prevalence of ARGs was determined for four ranges: 0, 1–8, 12–20, and 21–28. (**B**) Frequency of mutations in *gyrA* and *parC*.

The specific ARG information of *Salmonella* strains is shown in Figure 3. Firstly, among them, the most common ARGs are *gyrA* (66.67% (38/57)) and *parC* (61.4% (35/57)). Moreover, the proportion of *Salmonella* without ARGs was 10.53% (6/57), while the proportion of *Salmonella* with 1–8, 12–20, and 21–28 ARGs was 56.14% (32/57), 24.56% (14/57), and 8.78% (5/57), respectively (Figure 4A). Overall, the proportion of *Salmonella* carrying two or more ARGs reached 52.63%. In particular, one *Salmonella* strain carrying 28 ARGs was detected. Furthermore, our mutation analysis of the ARGs revealed significant mutations at several key sites, as shown in Figure 4B: *gyrA* (D87G) (12.28%), *gyrA* (D87N) (21.05%), *gyrA* (D87Y) (3.51%), *gyrA* (S83F) (42.11%), *gyrA* (S83Y) (21.05%), *parC* (S80R) (26.32%), and *parC* (T57S) (61.40%). The most common *parC* and *gyrA* mutations were *parC* (T57S) and *gyrA* (S83F).

### 3.5. Whole-Genome SNP Analysis of Salmonella Strains

**Figure 5:**
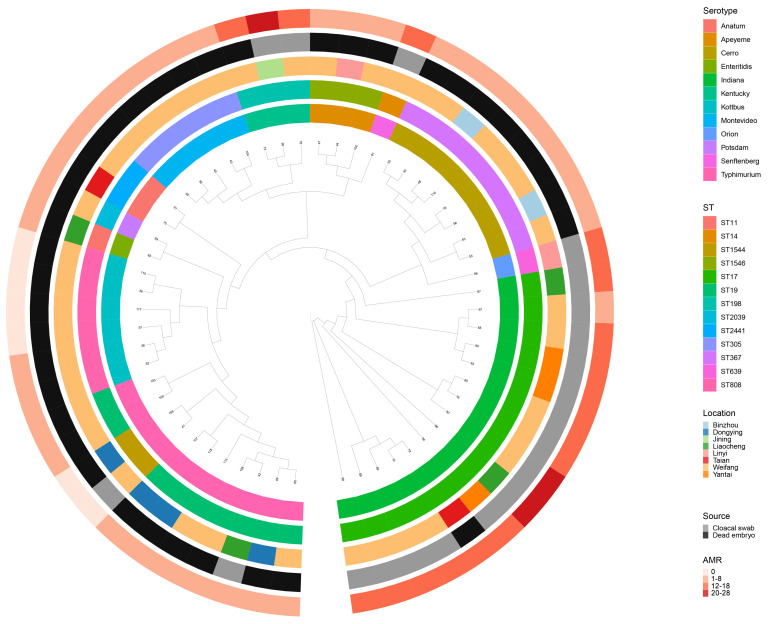
The phylogenomic relationships among 57 *Salmonella* strains. Different STs, locations, serotypes, sources, and antimicrobial resistance genes are indicated in different colors. The first circle represents the serotypes of isolated *Salmonella* strains, the second circle represents the STs of isolated *Salmonella* strains, the third circle represents the location of the strains, the fourth circle represents the sample types of strains, and the fifth circle represents the number of antimicrobial resistance genes, from the inside to the outside.

Whole-genome SNP analysis was used to investigate the deep phylogenetic relationship between the 57 *Salmonella* strains (Figure 5). The analysis showed that the isolates belonging to the same serotype or ST were closely related. In addition, the analysis indicates that the isolates from the same city are very closely related. Moreover, it can be seen that *Salmonella* belonging to the same serotype may originate from different cities, suggesting that there is a risk of cross-regional transmission of *Salmonella*. Furthermore, *S. Indiana* (ST17) carries more ARGs, and most of them are recovered from cloacal swabs. Whether this is related to serotype and STs needs further investigation.

## 4. Discussion

The widespread and inappropriate use of antimicrobial agents has accelerated the emergence and spread of AMR [22]. Although there have been reports of AMR *Salmonella* infections worldwide, the number of cases in developing countries is increasing significantly and at an alarming rate [23]. Clinical use of antibiotics is one of the main methods of treating *Salmonella*. The increase in farm density has also led to the emergence of MDR strains. As ducks are the natural reservoir of *Salmonella*, much attention is paid to *Salmonella* contamination in ducks. A total of 1116 environmental samples were collected from 31 duck farms investigated in Korea, and the positive rate of *Salmonella* ranged from 22.6% to 71% [24]. The positive rate of *Salmonella* in ducks in the Taiwan Province of China was 37.5% to 66.7% [25]. Reports from the United Kingdom show that the contamination rate of *Salmonella* in meat ducks is 29%, which is higher than that of chicken (5%) and other poultry meat (8%) [26]. The percentage of *Salmonella*-positive samples collected from the southwest Shandong Province and the province’s surrounding area was 21.74% (160/736) [27]. In this study, 13,585 samples of duck cloacal swabs and dead embryos collected from 278 duck farms in 11 cities of Shandong Province in 2023 were detected, and 57 strains of *Salmonella* were isolated and identified. The above studies indicate that meat ducks and their products may be an important vector of *Salmonella* disease in humans. Therefore, it is necessary to detect *Salmonella* periodically on duck farms.

With the rapid development of WGS, it can accurately predict the serotype of *Salmonella*. We then investigated and compared the genomic characterization and antimicrobial resistance genes by WGS. The MLST model based on WGS showed that there were 13 kinds of STs among 57 *Salmonella* strains. Among them, the highest proportion of STs is ST17, followed by ST19, which is similar to the previous research [4]. In addition, the dominant serotype in this study is *S. Indiana*, which is consistent with the previous report [4], but inconsistent with the report by Song et al. [27]. All *S. Indiana* strains exist in the form of ST17, and it has become a widespread and common non-typhoid serotype in China. The ST17 isolate is widely distributed in food, humans, animals, and the environment [28]. Previous reports indicate that the ST17 isolate has existed in China since 1959, experienced a major population expansion from 1980 to 2000, and began to decline in genetic diversity in 2011 [29]. In addition, the ST17 isolate from China is genetically similar to the isolates from the UK, the United States, South Korea, and Thailand, which may be the result of global transmission [30]. It is worth noting that we have reported the occurrence of ST305 for the first time, and its corresponding serotype is *S. Montevideo*, which has previously been reported to be closely related to cattle *Salmonella* [31]. In addition, to the best of our knowledge, this is the first time that ST808 (*S. Kottbus*) has been detected in duck samples, and the previous report was originally isolated from human and pork samples [32], which indicates that *S. Kottbus* is likely to spread to ducks by cross-species transmission, which should arouse our attention.

The results of the serotype analysis showed that a total of 12 serotypes distributed among the 57 duck-derived *Salmonella* strains showed rich polymorphism with *S. Indiana*, while *S. Typhimurium* and *S. Cerro* were the dominant serotypes. *S. Indiana* strains have been reported in retail food (chicken, duck, pork, seafood, and other food sources) and the environment (urban wastewater, slaughterhouses, and food markets), especially in children under 6 years old with low immune function [4,30,33]. S *typhimurium* is one of the most common causes of human *Salmonella* infection and is the most common cause of acute gastroenteritis in the world [34]. This serotype has been isolated from animals, food, and humans, and the transmission between animals and humans via the food chain has been recorded [35,36]. Therefore, it is necessary to monitor the serotypes of *Salmonella*.

Antibiotic resistance phenotype can be directly related to homologous ARGs, and we found 60 ARG types in this study. The proportion of *Salmonella* with 1–8 ARGs was 56.14%. Moreover, the most frequent ARGs are *gyrA* and *parC*. Mutation analysis showed that *parC* (T57S) had the highest mutation frequency, while previous reports proved that *parC* (T57s) was the most frequent mutation in quinone resistance-determining regions (QRDR) [37]. The strains containing both *gyrA* (D87N and S83F) and *parC* (T57S and S80I) gene mutations showed a high level of fluoroquinolone (FQs) resistance [38]. It has been reported that the *parC* (T57S) mutation is the most common of the QRDR mutations of *Salmonella* isolated from pet samples [39]. It is worth noting that the resistance to quinolones and fluoroquinolones (especially ciprofloxacin resistance) is an increasingly important problem in the world [40]. The main mechanism of mediating fluoroquinolone resistance is the mutation of QRDR and the existence of the plasmid-mediated quinolone resistance (PMQR) gene, which may help to select and promote the mutation of fluoroquinolone resistance genes [41,42,43].

## 5. Conclusions

In summary, 57 strains of *Salmonella* were collected from 13358 duck embryos and cloacal swabs were collected from 278 duck farms in 11 cities of China in 2023. *S. Indiana* and *S. Typhimurium* are the main serotypes, which are ST17 and ST19, respectively. *Salmonella* transmission also occurs through cloning and amplification in specific geographical areas and cross-species transmission. In addition, our data reveal a worrying trend of resistance to quinolones, which is also consistent with the holding of ARGs in different mutation types. It highlights the potential risk of foodborne infection caused by AMR *Salmonella*, and we should pay close and continuous attention to *Salmonella* isolated from animal-derived foods. Our research results fill the data gap in understanding the epidemiology of *Salmonella* in waterfowl in Shandong Province and provide baseline data for prioritizing interventions aimed at ensuring food safety and public health.

## Data Availability

The original contributions presented in the study are included in the article; further inquiries can be directed to the corresponding authors.
